# Pathophysiology of carotid-cavernous fistulas in vascular Ehlers-Danlos syndrome: a retrospective cohort and comprehensive review

**DOI:** 10.1186/s13023-018-0842-2

**Published:** 2018-06-25

**Authors:** Salma Adham, Denis Trystram, Juliette Albuisson, Valérie Domigo, Anne Legrand, Xavier Jeunemaitre, Michael Frank

**Affiliations:** 1 Hôpital Européen Georges Pompidou, Département de Génétique, Centre de Référence des Maladies Vasculaires Rares, Hôpital Européen Georges Pompidou, AP-HP, 20-40 rue Leblanc, 75908 Paris Cedex 15, France; 2 Sorbonne Paris Cité, Faculté de Médecine, Université Paris Descartes, Paris, France; 3 Service d’imagerie morphologique et fonctionnelle, Centre hospitalier Sainte-Anne, Paris, France; 4 INSERM, U894, IMA-BRAIN, DHU NeuroVasc Sorbonne Paris Cité, Paris, France; 5 INSERM, U970, Paris centre de Recherche Cardiovasculaire – PARCC, Paris, France

**Keywords:** Vascular Ehlers-Danlos syndrome, Rare vascular disease, Genetics, Carotid-cavernous fistula, Neurology, Neuroophthalmology, Pathophysiology

## Abstract

**Background:**

Vascular Ehlers-Danlos syndrome (vEDS) is a rare condition characterized by connective tissue fragility. Direct spontaneous carotid-cavernous fistula (sCCF) is reportedly pathognomonic of vEDS. We conducted this study to understand the possible mechanisms of occurrence of sCCF in this subset of patients.

**Methods:**

We conducted a retrospective analysis of a monocentric vEDS cohort along with a literature review regarding sCCF in this condition.

**Results:**

Of 133 patients regularly followed in our centre between 2000 and 2017, 13 (9.8%) had a diagnosis of direct sCCF (92.3% female, median age 33.0 years, interquartile range (IQR) [26.0–39.5]). There were 7 Glycine missense and 6 splice-site variants but no variant leading to haploinsufficiency. The literature search identified 97 vEDS patients with direct sCCF (79.4% female, 7.2% sex not reported, median age 31.0 years, IQR [24.0–39.0]). Increased carotid circumferential wall stress, higher carotid distensibility and lower carotid intima-media thickness could contribute to a higher risk for direct sCCF in vEDS. There is no predictive factor for the occurrence of sCCF apart from female sex in vEDS.

**Conclusions:**

In vEDS, anatomical and pathophysiological features of the intra-cavernous internal carotid artery make it prone to shunting in the cavernous sinus, due either to a spontaneous rupture or to a spontaneous dissection with pseudoaneurysm formation. Direct sCCF in seemingly healthy young individuals should be highly suggestive of vEDS and prompt further investigation.

## Background

Vascular Ehlers-Danlos syndrome (vEDS, OMIM #130050) is a rare inherited connective tissue disorder with a prevalence estimated at 1/150000. It is an autosomal dominant transmitted disorder caused by diverse mutations in the *COL3A1* gene which encodes the pro-alpha1 chain of type III procollagen [1–3]. Vascular EDS is characterized by connective tissue fragility leading to life-threatening complications in seemingly healthy young adults. Easy bruising, thin and translucent skin, acrogeria and characteristic facial features may be a hint for clinicians. Intestinal and uterine fragility are classical features of vEDS, but generalized arterial fragility dominates the clinical picture with spontaneous dissections, aneurysms and arterial ruptures [2].

It is therefore unsurprising that vEDS has been associated with the development of spontaneous direct carotid-cavernous fistula (sCCF). Carotid-cavernous fistula (CCF) is an abnormal communication between the high-pressure carotid arterial system and the low-pressure cavernous venous system (CS). In direct CCF, internal carotid artery (ICA) wall disruption allows high-pressure blood to move into the CS, resulting in short-circuiting of the ICA arterial blood into the venous system of the CS. The most common symptoms are swelling of the eye, chemosis, pain, ophthalmoplegia and bruit [4]. Direct sCCF is rare in the general population as most direct fistulas are posttraumatic but seems to be more common in vEDS patients and especially in females.

We report in this article a series of 13 molecularly proven vEDS patients diagnosed with direct sCCF along with a review on literature regarding direct sCCF occurring in vEDS patients. The possible mechanisms of occurrence in this subset of patients are discussed.

## Methods

### Study population

Since 2000, all molecularly proven vEDS patients followed in our department are systematically monitored clinically, biologically and by means of imaging (Doppler ultrasound, MR-Angiogram and/or CT-scan) at regular time intervals.

Patients with vEDS regularly followed in the French National Reference Centre for rare vascular diseases between 2000 and 2017 were included in this retrospective cohort study. Probands and relatives were diagnosed with vEDS based on clinical presentation, physical examinations, radiological findings and confirmation of a pathogenic variant in the *COL3A1* gene. Genetic testing was performed in compliance with the French legislation on genetic diagnostic tests (French bioethics law no. 2004–800) and written informed consent was given by all patients. A retrospective review of medical records for this study was approved by an ethical standards committee, the Comité de Protection des Personnes Ile-de-France 2 (IRB 00001072). Medical history was reviewed to identify patients suffering from direct sCCF. Patients’ characteristics were reported only for the patients with fistulas.

### Statistical analysis

A statistical analysis was performed with the XLStat software (Addinsoft, version 2017.4). To analyse patients’ characteristics, time-independent qualitative variables were compared using Chi-square test or Fisher’s exact test whenever the count was insufficient. Quantitative variables were reported using parametric descriptive statistics. A *P* value < 0.05 was considered significant.

### Literature review

A literature search was performed via computer literature searches. Articles were identified through a systematic PubMed search and by a review of their respective references. Available articles in English or in French regarding direct sCCF in vEDS patients were included.

## Results

### Study population

Since 2000, our centre has followed 148 molecularly proven vEDS patients. We identified 15 individuals either with variants leading to haploinsufficiency (*n* = 12) or with non-Glycine missense variants within the triple helix and non-Glycine missense variants or in-frame insertions-deletions in the N- or C-terminal part of the protein (*n* = 3). None of these 15 patients had a history of direct sCCF. Of 133 patients known to have either Glycine missense or splice-site and in-frame insertions-deletions variants in the *COL3A1* gene, there were 79 (59.4%) females, 90 (67.7%) Glycine missense variants, 55 (41.4%) patients with a history of digestive event and 66 (49.6%) patients with a history of acute arterial event.

Among these 133 patients, we identified 13 (9.8%) individuals who suffered from direct sCCF diagnosed between January 1985 and September 2014 (Table 1). There were 7 (53.8%) Glycine missense variants and 6 (46.2%) splice-site variants (OR 0.52 with a 95% confidence interval (CI) [0.14, 2.02], *P* 0.349). There were 12 (92.3%) females and 1 (7.7%) male (OR 0.11 with a 95% confidence interval (CI) [0.002–0.760], *P* 0.014). The median age at molecular diagnosis was 32.0 years, interquartile range (IQR) [28.0–39.0]. The median age at time of direct sCCF diagnosis was 33.0 years, IQR [26.0–39.5].

**Table 1:** Case series

Case no.	Age (years)	Sex	sCFF side	Treatment	Outcome	IMT (mm)	*COL3A1* variant cDNA	Protein	Type
1	34	F	Left	ICA sacrifice	Alive	0.383	c.665G>A	p.(Gly222Asp)	Glycine
2	30	F	Right	CCF closure (embolization)	Alive	0.354	c.2735G>A	p.(Gly912Asp)	Glycine
3	13	F	Left	CCF closure (embolization)	Alive	0.339	c.951+1G>A	p.?	Splice
4	21	F	Left	CCF closure (embolization)	Alive	0.372	c.2553+1G>A	p.?	Splice
5	33	F	Right	CCF closure (embolization)	Alive	0.394	c.1662+1G>A	p.?	Splice
6	42	F	Left	ICA sacrifice	Alive	0.387	c.3441_3485dup	p.(Lys1150_Gly1164dup)	Duplication
7	27 31	F	Right Left	CCF closure (embolization)	Died aged 38	0.319	c.2222G>A	p.(Gly741Asp)	Glycine
8	38	F	Right	CCF closure (embolization)	Alive	0.503	c.2095G>C	p.(Gly699Arg)	Glycine
9	25	F	Right	CCF closure (embolization)	Alive	NR	c.898-1G>C	p.?	Splice
10	36	F	Right	CCF closure (embolization)	Alive	0.435	c.2293G>C	p.(Gly252Arg)	Glycine
11	28	M	Right	CCF closure (embolization)	Alive	NR	c.[1347+1G>A];[=]	p.?	Splice
12	49	F	Right	CCF closure (embolization)	Alive	0.417	c.755G>T	p.(Gly252Val)	Glycine
13	41	F	Left	Died before treatment	Died aged 41	NR	c.826G>A	p.(Gly276Ser)	Glycine
Case no.	Age at molecular diagnosis (years)		1st vascular event	Age at 1st vascular event (years)	Acute arterial events (other than sCCF)	1st digestive event	Age at 1st digestive event (years)		
1	38		sCCF	34	Yes	Incisional hernia	32		
2	35		sCCF	30	Yes	–	–		
3	32		sCCF	13	Yes	Postoperative adhesive intestinal obstruction	25		
4	22		sCCF	21	No	–	–		
5	28		sCCF	33	Yes	Intestinal perforation	21		
6	38		AR	33	Yes	–	–		
7	28		sCCF	27	Yes	Diverticulitis	26		
8	32		sCCF	38	Yes	–	–		
9	25		sCCF	25	Yes	Occlusion on paresis	25		
10	45		AVF	24	Yes	–	–		
11	28		sCCF	28	No	–	–		
12	49		AR	48	Yes	Sigmoiditis	48		
13	40		ADO	21	Yes	–	–		

*sCCF* spontaneous carotid-cavernous fistula, *F* female, *ICA* internal carotid artery, *IMT* intima-media thickness, *M* male, *NR* not reported, *ADO* arterial dissection and occlusion, *AR* arterial rupture, *AVF* arteriovenous fistula

Direct sCCF was the first symptomatic vascular complication reported in 9 (69,2%) patients (cases 1 to 5, 7 to 9 and 11). Of 13 patients, 5 (38.5%) had already been diagnosed with vEDS 44.0 months, IQR [7.0–60.5], before the occurrence of the fistula (cases 5, 6, 8, 12 and 13). Occurrence of a direct sCCF led to vEDS molecular diagnosis in less than 15 months in 4 (30.8%) patients (cases 4, 7, 9 and 11, median diagnosis time 7.5 months, IQR [6.0–9.0]). Molecular diagnosis of vEDS was delayed to 79.5 months, IQR [49.5–166.5], after the sCCF in the 4 (30.8%) remaining patients (cases 1 to 3, case 10).

Data regarding the previous ICA status was not available in 4 (30.8%) patients (cases 2, 4, 8 and 10). No previously known aneurysm or dissection of the ICA was found in the medical history or in the radiological reports of 3 (23.0%) patients (cases 3, 9 and 13). Imaging during the systematic follow-up described a dysplastic aspect of the petrous ICA (probably secondary to a dissection) a few months before the ipsilateral sCCF diagnosis in one patient (case 6). Imaging at the time of the sCCF described an aspect of ICA dissection ipsilateral to the fistula in 4 (30.8%) patients (cases 1, 5, 7 and 12). Another patient (case 11) was diagnosed with cavernous ICA dissection and petrous aneurysm rupture leading to an ipsilateral fistula a few weeks later. Overall, there was no aneurysm or dissection of the contralateral ICA or in the other cerebral arteries reported on imaging at time of sCCF imaging diagnosis.

A patient (case 7) developed a sCCF during pregnancy, a second patient (case 2) developed a sCCF 2 years after her second pregnancy, a third one (case 10) developed a sCCF 3 years after her first pregnancy and a fourth patient (case 1) developed a sCCF 5 years after her second pregnancy. The patient reported as case 12 developed a sCCF 12 years after a second pregnancy. The median age of pregnancy in these 9 patients was 29.0 years, IQR [28.0–30.0], and the median age of occurrence of their fistula was 30.0 years, IQR [25.0–30.0] (*P* 0.888).

Most patients (76.9%) were treated by selective occlusion of the fistula (cases 2 to 5 and 7 to 12). Sacrifice of the ICA to achieve occlusion of the sCCF was necessary in 2 (15.4%) patients (cases 1 and 6), and one (7.7%) patient (case 13) died before treatment of the sCCF. A contralateral sCCF occurred in a patient (case 7) 5 years after the first sCCF embolization. All patients are still alive, except for case 7 who died of a ruptured aortoiliac dissection 11 years after the first sCCF embolization and 6 years after the contralateral embolization, and case 13 who died of a ruptured splenic dissection 1 month after sCCF diagnosis.

### Literature review

A literature search was performed on July, 2017, using PubMed. Abstracts were reviewed by a single reviewer and full-text articles were obtained for the studies meeting the eligibility criteria. Reference lists were also examined for additional relevant studies not identified through the initial search. Inclusion criteria were English-language and French-language full-text publications published since the 1950s, regarding vEDS patients diagnosed with sCCF.

We identified 51 articles [4–54], mainly case reports, describing 101 direct CCF in 97 vEDS patients (Table 2). There were 77 (79.4%) females and 13 (13.4%) males. Sex was not reported for 7 (7.2%) individuals [21, 40]. Median age at direct CCF diagnosis was 31.0 years, IQR [24.0–39.0] (one missing value [34]). Pepin et al. reported an overall mean age of 30.9 years in 27 patients. Five (5.2%) patients [18, 40, 46, 47, 50] experienced a direct sCCF recurrence on the contralateral ICA 2.5 years, IQR [2.0–3.5] after the first fistula. Of 97 patients, 4 (4.1, 75.0% male) were diagnosed either with a direct CCF following minor trauma [7, 11, 14] or with an iatrogenic direct CCF after placement of a Fogarty balloon into the cavernous segment of the ICA [28].

**Table 2:** Literature review

Authors	Age (years)	Sex	CCF side and type	*COL3A1* variant
François P et al. Bull Soc Ophtalmol Fr 1955 [16]	52	F	Right – NR	NR
Graf CJ et al. Arch Neurol 1965 [19]	24	F	Left – Direct	NR
	26	M	Left – Direct	NR
	37	F	Left – Direct	NR
Bannerman RM et al. Br Med J 1967 [6]	37	F	Left – Direct	NR
Schoolman A et al. J Neurosurg 1967 [47]	39 3 weeks later	F	Left – Direct Right – Direct	NR
Julien I et al. Presse Med 1971 [25]	50	F	Right – Direct	NR
Maugery G et al. Bull Soc Ophtalmol Fr 1972 [37]	17	F	Left – Direct	NR
Farley MK et al. Ophthalmology 1983 [12]	22	F	Left – Direct	NR
Guiolet M et al. Bull Soc Ophtalmol 1984 [20]	31	F	Left – Direct	NR
Dany F et al. J Mal Vasc 1986 [9]	29	F	Right – Direct	NR
Lach B et al. J Neurosurg 1987 [32]	43	F	Left – Direct	NR
Peaceman AM et al. Obstet Gynecol 1987 [42]	27	F	NR – NR	NR
Fox R et al. J Neurol Neurosurg Psychiatry 1988 [15]	20	M	Left – Direct	NR
Halbach VV et al. Neurosurgery 1990 [21]	22	F	Left – Direct	NR
	19	NR	NR – Direct	NR
	22	F	Left – Direct	NR
	24	NR	Right – Direct	NR
	39	F	Left – Direct	NR
	49	F	Right – Direct	NR
Pope FM et al. Br J Neurosurg 1991 [44]	22	M	Left – NR	NR
	25	F	Left – NR	NR
	45	F	Right – NR	NR
	27	F	Left – NR	NR
Schievink WI et al. J Neurosurg 1991 [46]	17 20	F	Left – Direct Right – Direct	NR
Kashiwagi S et al. Surg Neurol 1993 [27]	22	M	Left – Direct	NR
North NK et al. Ann Neurol 1995 [40]	17	F	NR – NR	NR
	27	NR	NR – NR	NR
	32	NR	NR – NR	NR
	24	NR	NR – NR	NR
	28	NR	NR – NR	NR
	28 33	NR	NR – NR Contralateral – NR	NR
Forlodou P et al. Neuroradiology 1996 [14]	40	F	Right – Traumatic, direct	NR
Debrun GM et al. Surg Neurol 1996 [10]	39	F	Right – NR	NR
	39	F	Left – NR	NR
	39	F	Left – NR	NR
Pollack JS et al. Arch Ophthalmol 1997 [43]	33	F	Right – Direct	NR
Bashir Q et al. Interv Neuroradiol 1999 [7]	50	F	Left – Traumatic, direct	NR
Koh J-H et al. Circulation 1999 [30]	21	F	Left – Direct	NR
Horowitz MB et al. AJNR Am J Neuroradiol 2000 [23]	18	F	Left – Direct	NR
	40	F	Right – Direct	NR
Kanner AK et al. J Neurosurg 2000 [26]	33	F	Left – Direct	NR
Chuman H et al. J Neuroophthalmol 2002 [8]	57	M	Left – Direct	IVS8+5G>A
	48	F	Left – Direct	c.544G>S
Favrole P et al. La Lettre du Neurologue 2003 [13]	32	F	Left – Direct	NR
Mitsuhashi T et al. Neurol Med Chir (Tokyo) 2004 [38]	30	F	Right – Direct	NR
Desal HA et al. Neuroradiology 2005 [11]	48	F	Left – Traumatic	NR
Jindal R et al. Eur J Vasc Endovasc Surg Extra 2005 [24]	30	F	Right – Direct (pregnancy)	NR
Hollands JK et al. Neuroradiology 2006 [22]	34	F	Left – Direct	NR
Usinskiene J et al. Cardiovasc Intervent Radiol 2006 [49]	25	F	Right – Direct	NR
Van Overmeire O et al. Interv Neuroradiol 2006 [4]	45	F	Left – Direct	NR
Yang JH et al. J Korean Med Sci 2007 [51]	48	F	Left – Direct	c.2195G>T
	36	F	Left – Direct	c.3221_3235dup
Zilocchi M et al. AJR Am J Roentgenol 2007 [52]	20	F	NR – Direct	NR
Drera B et al. J Derm Sci 2011 [53]	32	F	NR – NR	c.1655A>G
Khan A et al. J Neurointervent Surg 2012 [28]	20	M	Right – Traumatic, direct	NR
Mammen S et al. Neurol India 2012 [35]	27	F	Right – Direct	NR
Gauthier CE et al. J Mal Vasc 2013 [17]	25	F	NR – Direct	NR
Girardin M et al. Ann Dermatol Venereol 2013 [18]	32 34	F	Right – Direct Left – Direct	p.(Gly912Asp)
Wang Q et al. J Neurol Surg A Cent Eur Neurosurg 2013 [50]	37 39	F	Left – Direct Right – Direct	NR
Ananth AL et al. Semin Pediatr Neurol 2014 [5]	17	F	Left – Direct	c.3158G>A
Kim JG et al. J Korean Neurosurg Soc 2014 [29]	46	F	Right – Direct	c.1662+1G>A
Pepin M et al. NEJM 2014 [54]	Mean age 30.9	22F, 5 M	NR – NR	NR
Tanaka T et al. Neurol Med Chir (Tokyo) 2014 [48]	37	F	Left – Direct	NR
Kojima A et al. Interv Neuroradiol 2015 [31]	59	F	Right – Direct	NR
Linfante I et al. J Neurointervent Surg 2015 [34]	NR	M	Right – Direct	NR
Liang JW et al. Neurology 2016 [33]	25	F	Right – Direct	NR
Nakagawa I et al. J NeuroInterv Surg 2016 [39]	24	M	Right – Direct	NR
Ohlsson M et al. Neuroradiology 2016 [41]	33	F	Right – Direct	NR
Samaraweera APR et al. Neurology 2016 [45]	40	F	Right – Direct	c.1347+1G>A
Masson-Roy J et al. Can J Neurol Sci 2017 [36]	39	F	Left – Direct	NR

*CCF* carotid-cavernous fistula, *F* female, *M* male, *NR* not reported

Death was reported for 20 (20.6%) patients [4, 6, 7, 9–12, 19, 21, 23, 24, 26, 32, 43, 44, 47, 49, 51], of which 14 (70.0%) died in the course of sCCF management [4, 7, 9, 11, 12, 19, 23, 24, 32, 43, 44, 49, 51]. *COL3A1* variants were reported in 9 patients. There were 5 Glycine missense variants [5, 8, 18, 51, 53] and 4 splice-site variants [8, 29, 45, 51]. In the other patients, vEDS diagnosis was made either on relevant clinical examination, imaging and medical history [6, 7, 9, 10, 12, 15, 19, 27, 28, 30, 31, 33, 34, 39, 42, 46–50, 52], histological findings [6, 9, 24, 27, 32, 43, 44, 47] or on fibroblast culture [8, 15, 36, 38, 40, 43, 44]. Imaging described marked tortuosity or dysplastic aspect or ectasia of the ICA segment in the neck [6, 10, 19, 26, 39, 49], sometimes associated with an aneurysm rupture of the intracranial ICA segment into the CS [8, 15, 19]. Aneurysms of the ICA without rupture were also reported [12, 30, 51], sometimes associated with images of dissection [33, 46, 52].

## Discussion

Classification of CCF is based on aetiology (traumatic versus spontaneous), blood flow velocity (high versus low) and anatomy (direct versus dural, or ICA versus ECA). According to the Barrow classification, there are 4 types of CCF [55]. Type A or direct CCF is a high-flow direct shunt due to a posttraumatic or ruptured aneurysm which results in short-circuiting of the ICA arterial blood into the venous system of the CS. Type B, C and D are low-flow lesions and grouped under the common definition of dural or indirect CCF. In the general population, direct CCF represents 75% of all CCF and is mostly posttraumatic (0.2–0.3% of craniofacial trauma) [56] or associated with surgical complications, while spontaneous dural (indirect) low-flow CCF are mostly found in postmenopausal hypertensive females [57]. Direct sCCF only accounts for 10% of all spontaneous CCF [58]. The intracavernous ICA is prone to spontaneous shunting due to the amount and direction of ligaments in relation to the adventitia, the conformation and mobility of the siphon (horizontal segment), and the inextensibility of the dura [59, 60]. Direct sCCF can be found in patients with intracavernous ICA aneurysms, but only 2 to 9% of intracavernous ICA aneurysms are complicated with a direct CCF [61]. The rupture of a persistent trigeminal artery-associated aneurysm has also been associated with the development of CCF [62, 63]. Though direct sCCF seems to be rare in healthy individuals, it appears to be a significant complication in patients with collagenopathies such as vEDS [40, 46, 64]. Spontaneous CCF does not seem to associate with other rare vascular diseases such as Marfan disease or Loeys-Dietz syndrome [65].

Vascular EDS is a rare heritable disease mainly characterized by vascular manifestations such as rupture, dissection or aneurysm affecting large or medium-sized arteries. Cervicocerebral arteries can be affected with disorders such as intracranial aneurysm, carotid artery dissection or direct CCF [64–66]. In our tertiary referral centre, subjects with direct CCF represent 9.8% of vEDS patients and all fistulas were spontaneous. Posttraumatic direct CCF seems to be rare in vEDS patients and occurs mostly in males [7, 11, 14].

The median age of our patients at direct sCCF diagnosis, 33.0 years, IQR [26.0–39.5], is quite similar to previous data reported in vEDS population [46, 54, 64], and considerably lower than patients free from vEDS who experience CCF [55]. These results are also concordant with the median age (31.0 years, IQR [24.0–39.0]) of the patients identified in the literature review. In our series, it is remarkable that 9 patients (69.2%) experienced a direct sCCF as their first vascular event, prompting further investigations for vEDS molecular diagnosis in 4 patients only. Notably, direct sCCF has recently been included in the 2017 classification for vEDS as a major diagnosis criterion [2]. As direct sCCF in young patients seems to be pathognomonic of vEDS, genetic testing of the *COL3A1* gene should be mandatory in such patients.

The type of pathogenic variant identified within the *COL3A1* gene encoding the pro-alpha1 chain of type III procollagen in vEDS patients who experienced sCCF is another interesting finding. The natural course of vEDS depends on the type of *COL3A1* variant, and there are more severe clinical and phenotypical presentations in Glycine missense and splice-site variants than in variants leading to haploinsufficiency [67]. Our 13 patients presented either with heterozygous Glycine missense or splice-site variants of exons encoding a triple helix sequence. The literature review identified 9 patients with available pathogenic variants within the *COL3A1* gene, and all were unsurprisingly either Glycine missense [5, 8, 18, 51, 53] or splice-site variants [8, 29, 45, 51]. Therefore, it seems reasonable to hypothesize there might be a strong association between these variants and the occurrence of sCCF, while haploinsufficiency does not associate with fistulas. However, the total number of pathogenic variants identified in our patients or reported in the literature (22/97, 22.7%) is too small to confirm this genotype-phenotype correlation. As the genetic test is now mandatory to confirm clinical diagnosis, the presence of pathogenic *COL3A1* variants should be systematically reported in future articles related to sCCF.

In vEDS patients, spontaneous rupture of a tortuous ICA emerging from the petrous bone into the CS can result in a direct fistula with [8, 15, 19] or without [6, 10, 19, 26, 39, 49] a previous known intracranial ICA aneurysm. Another mechanism could be a spontaneous dissection of the ICA with pseudoaneurysm formation [68]. Spontaneous ICA dissection leading to direct sCCF is described in 5 of our patients (cases 1, 6, 7, 11 and 12) and in the literature review [33, 46, 52]. Intracranial arteries have a histological constitution that reportedly differs from diameter-matched extracranial arteries. They have a decreased amount of elastic fibers in the media and adventitia layers, along with thinner collagen fibers and no external elastic lamina [69]. It is well known that vEDS leads to vascular complications due to structural weakness of the artery wall secondary to type III collagen defects. Therefore, arterial ruptures can also occur on previously non-aneurysmal arteries as we found in 3 patients (cases 5, 9 and 13). Notably, direct sCCF in vEDS patients do not systematically result from aneurysm rupture or dissection of the cavernous ICA and cannot be exclusively described as a complication of an aneurysm or dissection of the cavernous ICA. An explanation for such spontaneous ruptures in seemingly healthy arteries can be found in Boutouyrie et al. [70] who demonstrated an increased carotid circumferential wall stress and a higher carotid distensibility in vEDS patients compared with healthy individuals. Moreover, vEDS patients are known to have lower IMT [70] and lower diastolic-systolic arterial stiffening than healthy control individuals [71]. Impairment of type III collagen generates a lower carotid stiffness and an abnormally low IMT, leading to higher wall stress and risk of arterial rupture. As direct sCCF is probably due to a sudden increase of the ICA intraluminal pressure [72], the previous findings explain why vEDS are at high risk for direct sCCF.

The male to female ratios in our study (0.08) and literature review (0.15) stress the fact that female vEDS patients seem to be more at risk than males for direct sCCF. In comparison, there was a 0.79 male to female ratio in our non-sCCF vEDS patients. In addition to potential sex differences in IMT, sex differences in ICA anatomy could account for a higher prevalence of fistulas in females. Though gathering evidence in vEDS patients is challenging, inferring results from the general population is a first step in understanding the female predominance for sCCF in vEDS. Differences between males and females in various segments of the vascular system have been reported [73–75], as well as in intracranial arterial bifurcations and more specifically of the ICA [76, 77], suggesting the involvement of a physiological or an anatomical factor. First of all, sex differences in ICA can be found in the diameter of the parent ICA and its largest and smallest branches, with a significantly larger diameter in male vessels than in female vessels [77, 78]. Secondly, the study of ICA maximum wall shear stress shows a 50% higher stress in the female bifurcation compared with the male bifurcation [77]. As females have smaller ICA, their vessel walls are exposed to higher blood flow velocity, and this shear stress is reflected through a higher pressure drop in the female bifurcation compared with the male bifurcation. Vessel morphology and more specifically vessel curvature is also an interesting factor to take into account to determine hemodynamic patterns. Healthy females reportedly have higher mean curvature of their intracranial ICA than males (*P* 0.04) with a general tendency to a more curved ICA [79]. Only 20.5% of males have their peak curvature located within the CS, in comparison with as much as 45.1% of females. Therefore, we hypothesize that anatomical differences account for an irregular blood-flow pattern—which in turn accounts for the alteration of the arterial wall—due to higher shear forces in female intracranial ICA, and lead to more ICA ruptures and fistulas in female vEDS patients. Finally, evidence regarding lower IMT in healthy females compared to males can be found in the literature [80–82]: sex is significantly correlated with carotid artery IMT (*P* 0.001), as females have significantly lower IMT than age matched males [82]. However, due to the rarity of vEDS, there is no available evidence in the literature regarding IMT differences between male and female vEDS patients. Moreover, as carotid IMT is measured on the extracranial part of the carotid artery 1 cm beneath the bifurcation, the IMT profile of proximal ICA might not be translatable into structural fragility of the cavernous ICA. Nonetheless, assuming that IMT difference between healthy male and female individuals might also be found in vEDS males and females could account for the overwhelming prevalence of direct sCCF in female vEDS patients.

Another appealing and complementary hypothesis for the frequent occurrence of direct sCCF in vEDS females could be the decrease of wall thickness due to sex hormones, and in particular the role of oestrogens on arterial wall remodelling. An arteriovenous fistula model in rats reveals a significant decrease in wall thickness of the proximal and distal aorta in castrated male rats receiving oestrogens [83]. These results are consistent with the findings of a previous study: neointima formation is reduced by 60% in rats treated daily by oestrogens before and after balloon injury of carotid artery [84]. The role of oestrogens in spontaneous rupture of carotid-cavernous aneurysm has also been suspected during pregnancy in healthy females free from pregnancy-induced hypertension [85, 86]. We could not find evidence of oestrogen taking in oral-contraceptives for example in our female vEDS patients who experienced direct sCCF, but it is noteworthy that the median age of pregnancy in these patients is quite similar to the median age of occurrence of their sCCF. These results lead us to think that collagen fragility, IMT and anatomy differences are not the only factors predisposing to the rupture of arteries in vEDS females.

## Conclusions

Patients with vEDS have greater risk than healthy individuals to develop direct sCCF due to their fragile vessels walls. Direct sCCF may occur spontaneously by rupture of the ICA, secondary to the rupture of an ICA aneurysm or by dissection of the ICA. The type of variant at the *COL3A1* gene associates with the occurrence of fistulas, as only Glycine missense and splice-site variants were identified in our sCCF patients. Female vEDS patients evidently seem more at risk for direct sCCF than males. We hypothesize that this sex difference is essentially linked to ICA anatomical and possible IMT differences between females and males but might also be amplified by hormonal differences. These hypotheses call for further investigations in vEDS patients.

Finally, direct sCCF can be the first symptomatic event in a previously non-diagnosed vEDS individual: in one third of our patients, the occurrence of a fistula led to the molecular diagnosis of vEDS. As sCCF seems to be highly suggestive and possibly pathognomonic of vEDS, type III collagen deficiency should be suspected in young patients and especially in females presenting with a direct sCCF.

## Abbreviations

CCF: Carotid-cavernous fistula
CI: Confidence interval
CS: Cavernous sinus
ECA: External carotid artery
EDS: Ehlers-Danlos syndrome
ICA: Internal carotid artery
IMT: Intima-media thickness
IQR: Inter quartile range
sCCF: Spontaneous carotid-cavernous fistula
vEDS: Vascular Ehlers-Danlos syndrome
